# Analyzing the innate immunity of NIH hairless mice and the impact of gut microbial polymorphisms on *Listeria monocytogenes* infection

**DOI:** 10.18632/oncotarget.22051

**Published:** 2017-10-25

**Authors:** Zhong-Hao Ji, Wen-Zhi Ren, Wei Gao, Yang Hao, Wei Gao, Jian Chen, Fu-Shi Quan, Jin-Ping Hu, Bao Yuan

**Affiliations:** ^1^ Department of Laboratory Animals, College of Animal Sciences, Jilin University, Changchun 130062, Jilin, China

**Keywords:** NIH hairless mice, Listeria monocytogenes, gut microbiota, 16S rRNA, innate immunity

## Abstract

Spontaneous mutant hairless (HL) mice are often used to study hair growth and hair follicle development, and they often exhibit immune dysfunctions. *Listeria monocytogenes*, an important food-borne bacterium, has been used in animal models to study immune responses to infection. Herein, we analyzed the innate immunity of HL mice and the impact of gut microbial polymorphisms on *L. monocytogenes* infection. Compared to NIH mice, NIH HL mice were more susceptible to *L. monocytogenes*, as weight losses, mortality, bacterial load, and histopathological lesions were more severe; the decrease in monocytes may be an important underlying reason. The degree of spleen damage was reduced after co-housing, indicating that the host guides the gut microbiota to alleviate infection. High-throughput pyrosequencing of 16S rRNA demonstrated that gut microbiota composition differed between NIH HL and NIH mice. Infection with *L. monocytogenes* induced an increase in the number of bacteria belonging to the *Rikenellaceae* family and *Gammaproteobacteria* class, and decreased bacteria belonging to the *Clostridiales* class and *Lachnospiraceae* family. A substantial reduction in *Clostridiales* bacteria in infected HL mice may cause a serious infection. The *Mycoplasma* genus was present only in NIH HL mice and was, thus, considered a biomarker. The results of this study improve our understanding of the use of NIH HL mice as a good animal model of innate immune dysfunction.

## INTRODUCTION

The skin is the first immune barrier of the body. Spontaneous mutant hairless (HL) mice are used to study hair growth and hair follicle development [1]. It has complex functions and is closely linked to the immune, skin, and pigment systems [2]. In 2007, we found a mouse model with a spontaneous coat mutation (HL mice). The phenotype of this mouse was hairless, and hematoxylin and eosin (H&E) staining showed abnormal hair follicle structure and development cycles. Previously, we analyzed some of the biological characteristics of the mice [3] by obtaining the dorsal skin of female normal and HL mice 42 days after birth and subjecting the samples to RNA sequencing to examine gene-expression patterns in mutant mice [4], in order to determine their usefulness as an animal model.

*Listeria monocytogenes* is an important food-borne bacterium that has been used in important applications for studying the immune responses of animals to infection. To fight against *L. monocytogenes* infection, the host would be actively mobilized through both innate and adaptive immunity. Simultaneously, some immune-related genes might also play an important role in fighting infection [5, 6].

In contrast to humans, mice showed apparent tolerance when *L. monocytogenes* was administered orally to generate the infection model, which raises questions about the use of this animal model. The sensitivity to *L. monocytogenes* infection differs among different inbred mouse strains. Earlier studies demonstrated that the intravenous (i.v.) and intraperitoneal (i.p.) routes are important for studying *L. monocytogenes* infection; multiple genes interact with immune responses to infection, for example, the Hc locus on chromosome 2 [7], *Trail* [8], and *Lfa1* [9].

Germ-free mice can be easily recolonized with bacteria by placing a single mouse that has normal flora into a cage with other mice [10]. Since mice have the habit of swallowing feces, the intestinal microbes of mice tend to be consistent after co-housing different strains of mice in the same cage for about 4 weeks [11].

The genetic background and gut microbial composition have complex interactions [12] and no clear gut microbial polymorphisms have been shown to explain the difference in infection severity between NIH normal and NIH HL mice infected with *L. monocytogenes*. Therefore, we analyzed the innate immunity of NIH HL mice and the impact of gut microbial polymorphisms on *L. monocytogenes* infection.

## RESULTS

### HL mice are more susceptible to *L. monocytogenes* infection than NIH mice

**Figure 1:**
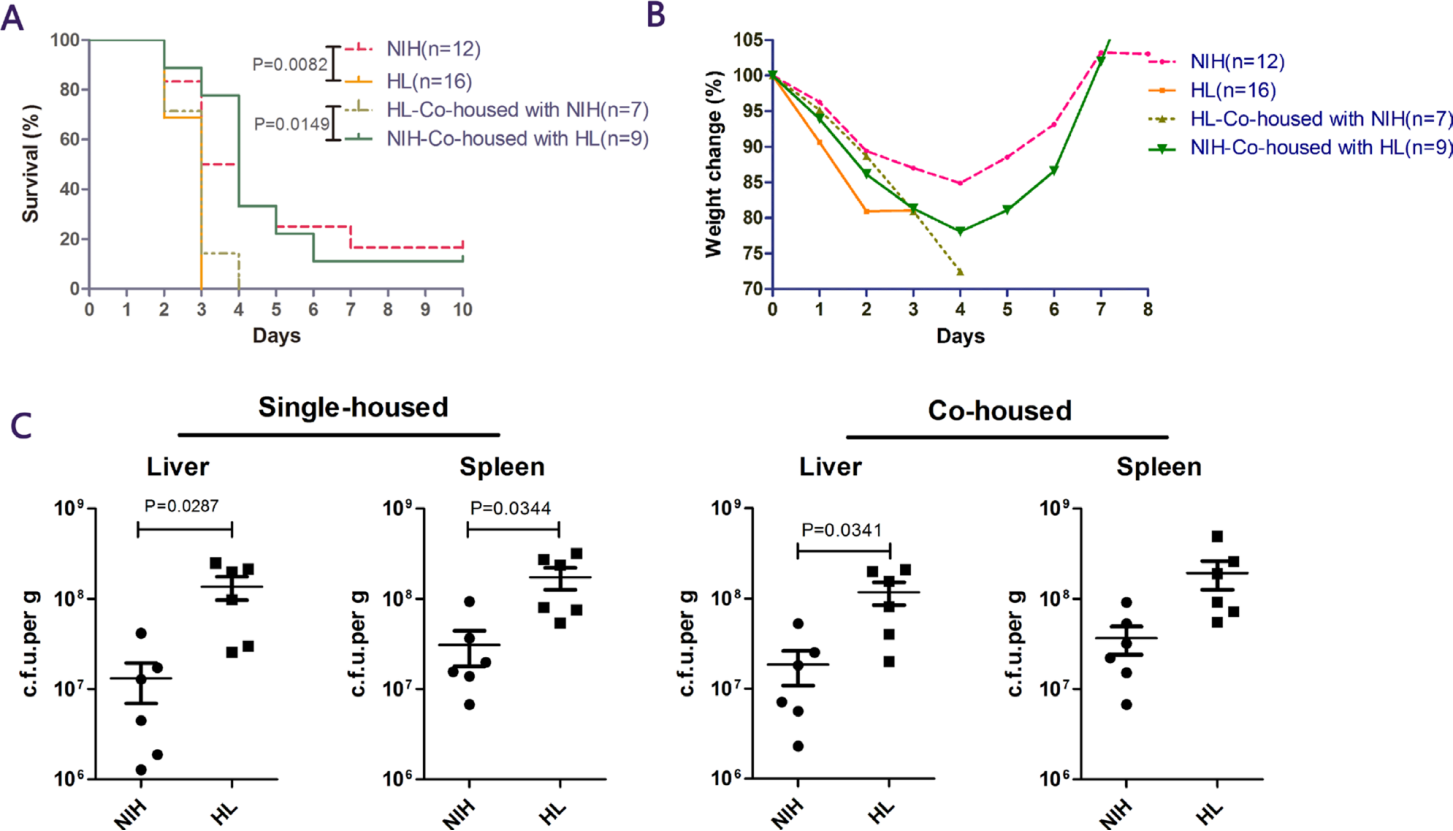
NIH hairless (HL) mice are susceptible to *Listeria* infection (**A**) NIH HL mice, normal (NIH) mice, HL mice co-housed with NIH mice, and NIH mice co-housed with HL mice. Mice were administered *L. monocytogenes* via i.p. injection, and survival was monitored daily for 10 days. (**B**) Proportion of weight loss. (**C**) Bacterial loads in the four groups of mice were determined in the liver and spleen on day 3.

In initial experiments, we analyzed the levels of resistance in four groups of mice (HL, NIH, HL co-housed with NIH, and NIH co-housed with HL) to an i.p. challenge with 2 × 106 colony-forming units (cfu) of *L. monocytogenes* strain 10403S. HL mice died within 2–3 days of infection, and 16.7% of NIH mice survived till the end of the experiment after infection. After co-housing both kinds of mice, HL mice succumbed to infection within 4 days, and no significant difference was observed in the mortality of NIH mice. (Figure 1A). Similarly, when mice began to die after infection, HL mice lost 20% of their body weight, while NIH mice lost only about 10% of their body weight, and weight loss was not noted after day 5 (Figure 1B). To understand the spread of bacteria in mice, the number of bacteria in two main susceptible organs was measured on day 3 after infection. The results showed that the bacterial burdens in HL mice were more severe than those in NIH mice, but the bacterial burdens in the spleen of co-housed mice were not significantly different (Figure 1C). These results showed that mutant HL mice are more susceptible to *L. monocytogenes* infection than NIH mice. Co-housing did not have a major impact on mice in terms of weight changes and survival, although the bacterial burden in the spleen of HL and NIH mice was not significantly different after co-housing.

### Histopathological lesions were more severe in HL mice than in NIH mice

**Figure 2:**
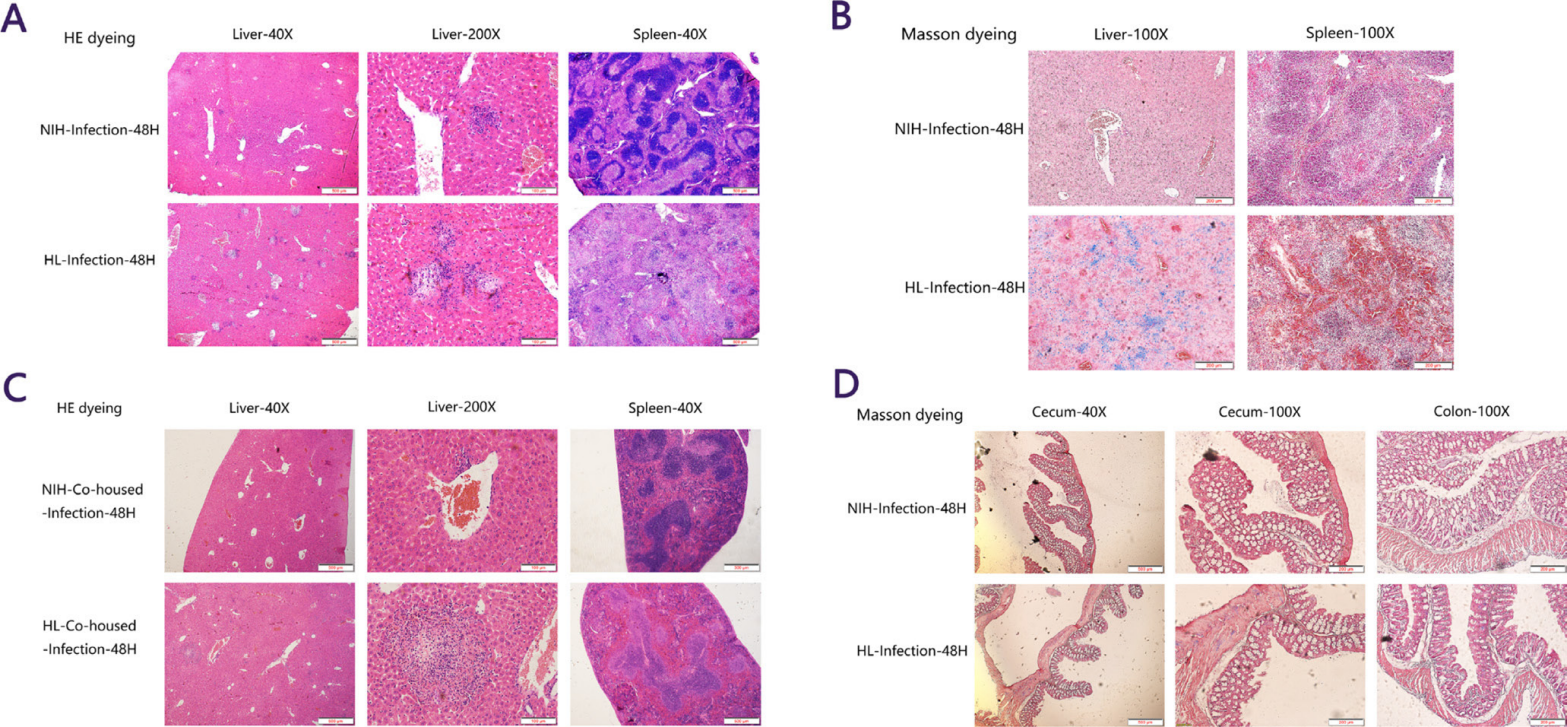
Histopathological lesions were more severe in HL mice than in NIH mice (**A**) In HL mice, large areas of inflammatory cells were found in the liver, the spleen tissue was severely damaged, and the boundaries between red and white pulp were blurred, compared to those in NIH mice. (**B**) Masson staining confirmed that the collagen fibers were stained blue during liver inflammation, and the bleeding areas in the splenic cord were stained orange red. (**C**) In HL-co-housed mice, a certain range of inflammatory cells was present in the liver, but the damage in the spleen was obviously relieved compared to that in HL mice. (**D**) Masson-stained cecum and colon sections of HL mice had more inflammatory lesions than did NIH mice.

To examine the pathological damage in organs and infiltration of inflammatory cells, H&E and Masson staining were performed. Consistent with previous results, large areas of inflammatory cells were found in the liver, the spleen tissue was severely damaged, and the boundaries between red and white pulp were blurred in HL mice, compared with those in NIH mice (Figure 2A). Masson staining also confirmed that the collagen fibers in the liver were stained blue due to an inflammatory response, and the bleeding areas in the splenic cord were stained orange red (Figure 2B). In HL-co-housed mice, a certain range of inflammatory cells was observed in the liver, but damage to the spleen was obviously reduced compared to that in HL mice (Figure 2C). In addition, Masson-stained cecum and colon sections of HL mice were found to have inflammatory lesions compared to that in NIH mice (Figure 2D). The negative control of Figure 2 was showed in Supplementary Figure 1. These results showed that histopathological lesions were more severe in HL mice than in NIH mice, and that co-housing can partially relieve spleen injury.

### Reduced monocyte recruitment in infected HL mice

**Figure 3:**
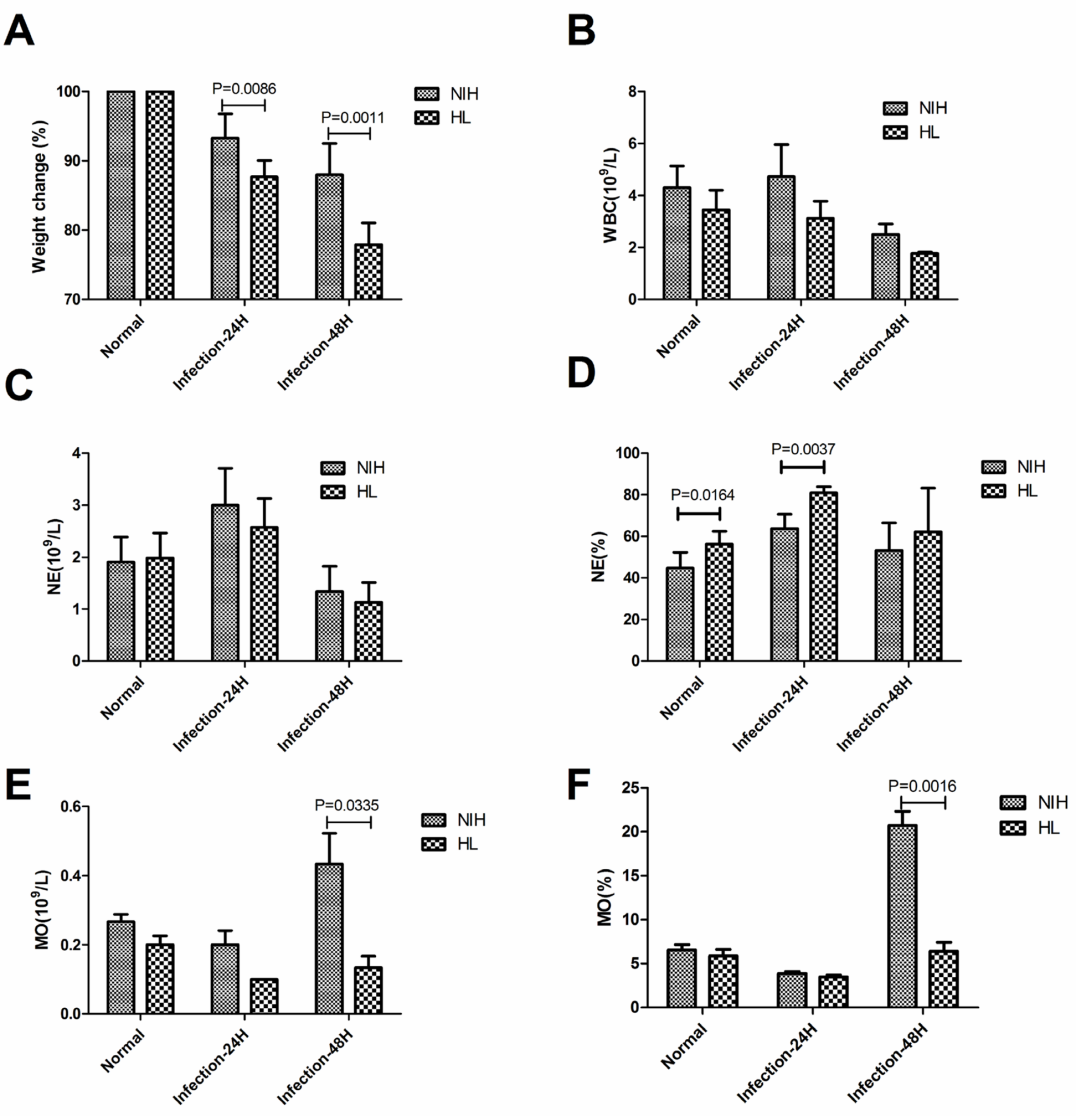
Reduced monocyte recruitment in infected HL mice (**A**) Proportion of weight loss after infection. (**B**) The number of white blood cells in infected HL and NIH mice was assessed using an automated hematology analyzer. (**C**) The number of neutrophils. (**D**) The proportion of neutrophils in white blood cells. (**E**) The number of monocytes. (**F**) The proportion of monocytes in white blood cells.

An automated hematology analyzer was used to analyze the quantities and proportions of monocytes and neutrophils. After infection, the weight of HL mice decreased significantly on days 1 and 2 compared to that of NIH mice (Figure 3A). No significant difference in the number of white blood cells was observed (Figure 3B). There was no significant difference in the number of neutrophils; however, in HL mice the proportion of neutrophils in the white blood cells was significantly higher on day 1 after infection than that in NIH mice (Figure 3C, 3D). Before infection, there were no significant differences in either the number of monocytes or their proportion in the white blood cells. However, after two days of infection, both indices were significantly lower in HL mice than in NIH mice (Figure 3E, 3F). These results showed that the monocyte levels were reduced significantly in HL mice at 48 h post-infection.

### Cytokine expression in the liver, spleen, and serum of infected HL and NIH mice

**Figure 4:**
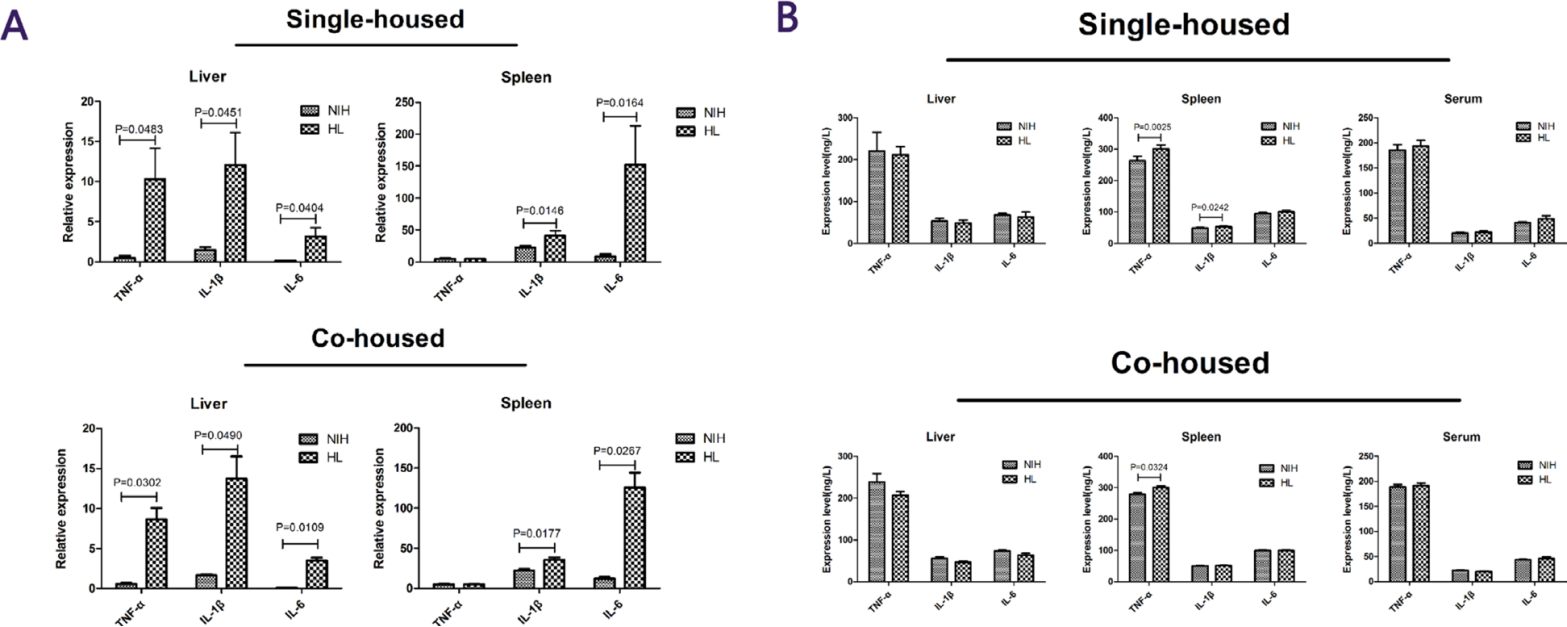
Cytokine levels in the liver, spleen, and serum of infected HL and NIH mice (**A**) The expression levels of TNF-α, IL-1β and IL-6 in the liver and spleen in single-housed mice and co-housed mice after infection. (**B**) The protein-expression levels of TNF-α, IL-1β, and IL-6 in the liver and spleen, in single-housed mice and co-housed mice after infection.

To investigate inflammatory changes in the organism after infection, the levels of inflammatory cytokines in serum, liver, and spleen were measured [13]. Quantitative results showed that in HL mice and HL-co-housed mice, the expression levels of *TNF-α*, *IL-1β*, and *IL-6* in the liver were significantly higher than those in the liver of NIH mice, and the expression levels of *IL-1β* and *IL-6* in the spleen were significantly higher than those in the spleen of NIH mice (Figure 4A). The enzyme-linked immunosorbent assay (ELISA) results showed that the expression levels of *TNF-α* and *IL-1β* in the liver were significantly increased in HL mice, compared to that in NIH mice. In HL-co-housed mice, only *TNF-α* protein expression levels in the liver were significantly increased, relative to that in NIH-co-housed mice. No other results showed a significant difference (Figure 4B). These results showed that inflammatory cytokines did not play a major role in infection with *L. monocytogenes*.

### Analysis of microbiome in the four groups of mice

**Figure 5:**
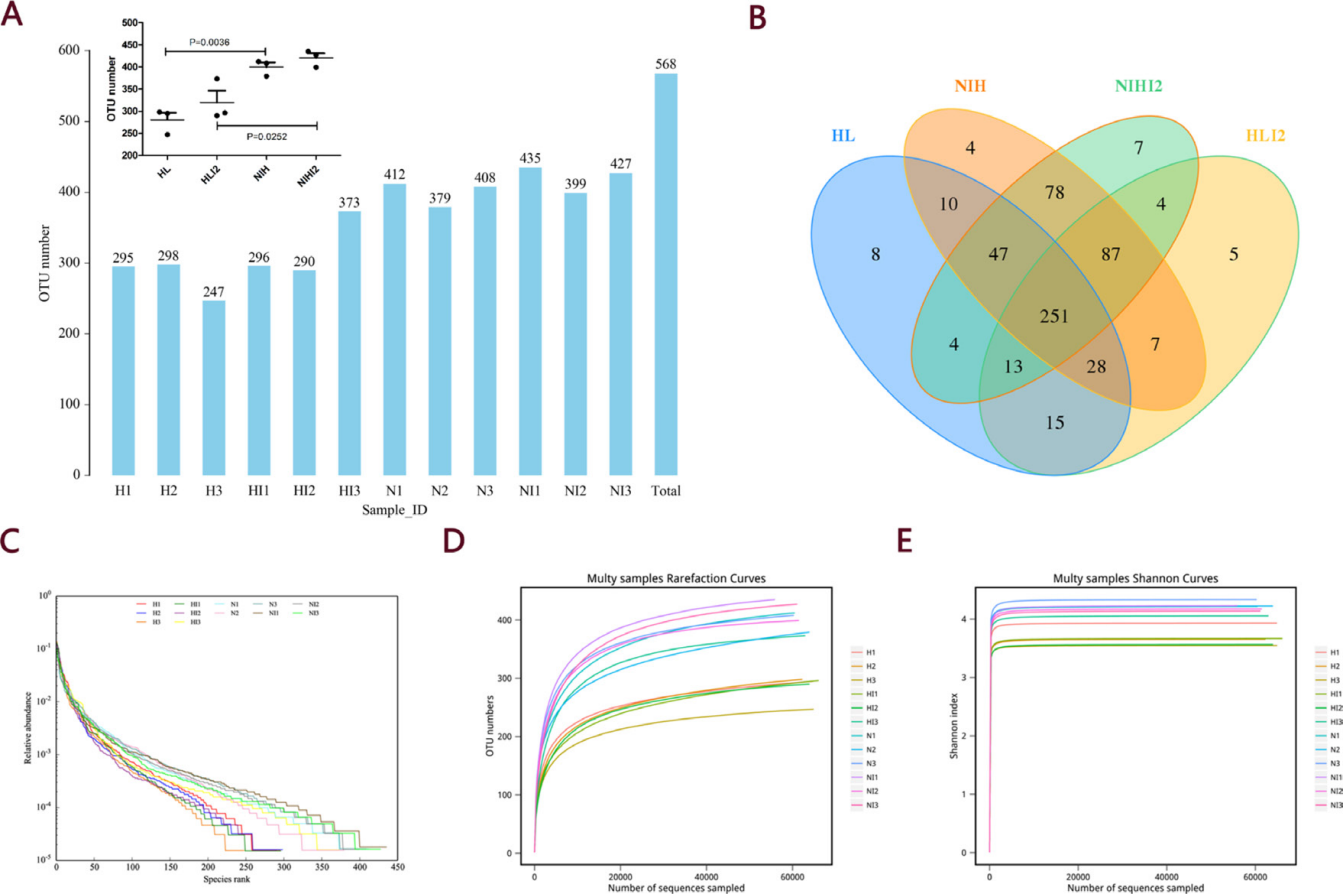
OTU analysis and alpha diversity analysis (**A**) OTU number. (**B**) Venn diagram of OTUs. (**C**) Curves showing the OTU rank. (**D**) Rarefaction curves. (**E**) Shannon index curves.

**Figure 6:**
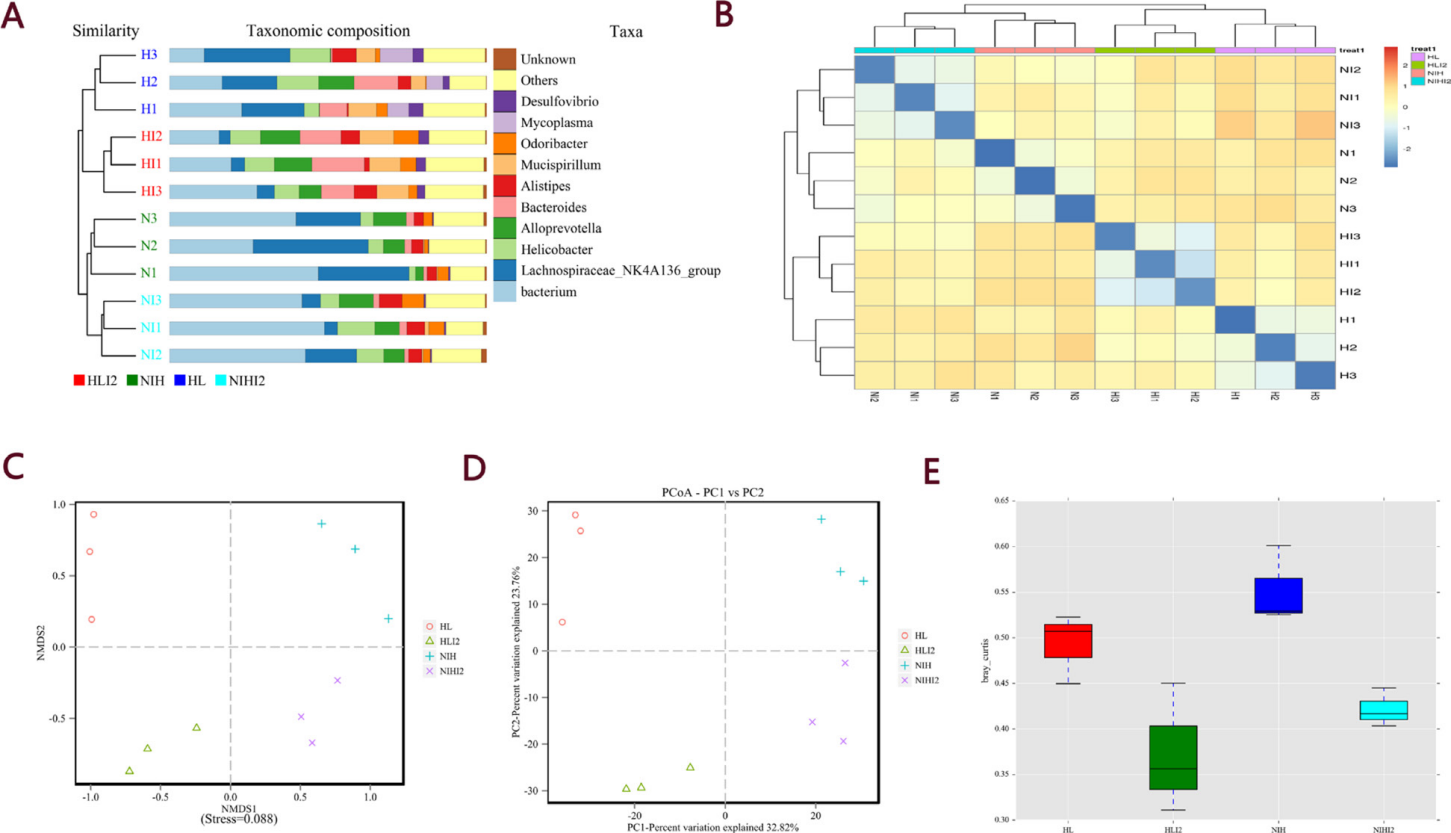
Beta analysis between groups based on the Bray–Curtis algorithm (**A**) UPGMA clustering tree and histogram combination drawing. (**B**) Heatmap. (**C**) NMDS plot. (**D**) PCoA plot. (**E**) Beta diversity box plot.

Before and after infection, the intestinal microbiota composition was assessed by sequencing the bacterial 16S rRNA V3 + V4 region [14]. Twelve samples were sequenced, and 960,392 pairs of reads were obtained. Double-end read splicing and filtering resulted in 813,531 clean tags, and each sample produced 80,033 clean tags (Supplementary Table 1). Using QIIME (version 1.8.0) UCLUST software based on 97% sequence similarity, the tags were clustered into operational taxonomic units (OTUs); no significant difference was noted in the number of OTUs between the normal and post-infection groups, while the number of OTUs in HL mice decreased markedly relative to those in NIH mice (Figure 5A). The Venn diagram of OTUs (Figure 5B) [15] and the OTU rank (Figure 5C), rarefaction (Figure 5D), and Shannon (Figure 5E) curves were calculated. Difference analysis between groups were based on the Bray–Curtis algorithm, which mainly included principal coordinates analysis (PCoA), non-metric multi-dimensional scaling (NMDS), and unweighted pair-group method with arithmetic mean (UPGMA). The clustering and PCoA diagrams are shown in Figure 6A–6E.

**Figure 7:**
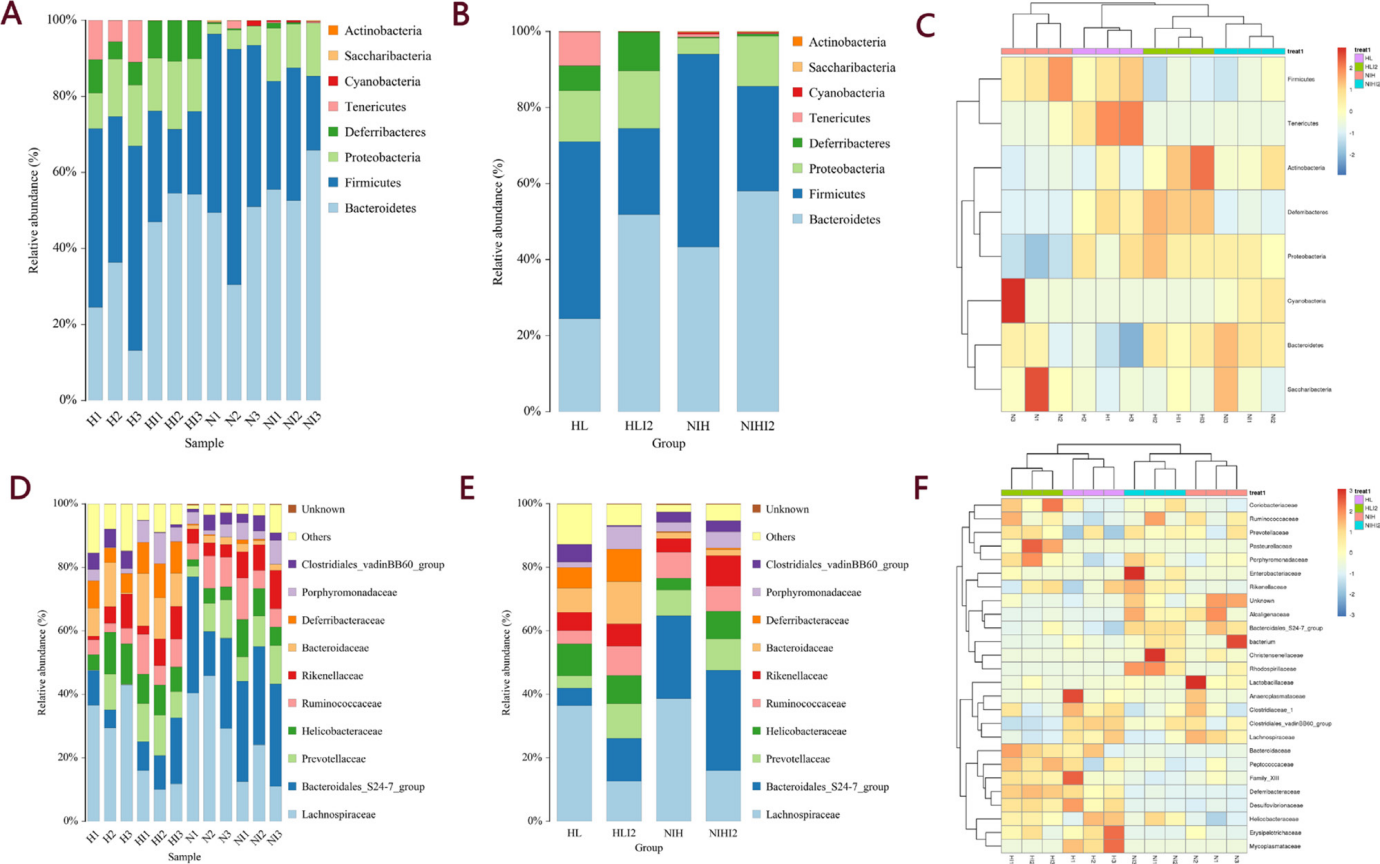
The community composition of each sample was calculated at various levels (**A**) Species-distribution histogram of different samples in a phylum. (**B**) Species-distribution histogram of different groups in a phylum. (**C**) The species abundance-clustering image in a phylum. (**D**) Species-distribution histogram of different samples in a family. (**E**) Species-distribution histogram of different groups in a family. (**F**) Species-abundance clustering image at the family level.

By comparing the representative sequence of OTUs with the microbial reference database, each OTU can be classified into a species. Simultaneously, the community composition of each sample was calculated at various taxonomic levels (phylum, class, order, family, genus, and species), and species-abundance tables at different classification levels were generated using QIIME software. The community structure of samples under different taxonomic levels was drawn using R language tools. As shown in Figure 7A–7B, the phylum-level analysis demonstrated that mice infected with *L. monocytogenes* showed a significantly decreased relative abundance of bacteria belonging to the Firmicutes phylum and an increased relative abundance of bacteria belonging to the Bacteroidetes phylum. Family-level analysis was performed to further study differences among each sample. As shown in Figure 7D–7E, the relative abundances of bacteria belonging to the Lachnospiraceae family decreased after infection, and the relative abundance of bacteria belonging to the *Bacteroidales* order decreased in HL mice (including HL normal and infected mice), as compared with that in NIH mice. Clustering of three samples in each group was consistent (Figure 7C, 7F).

**Figure 8:**
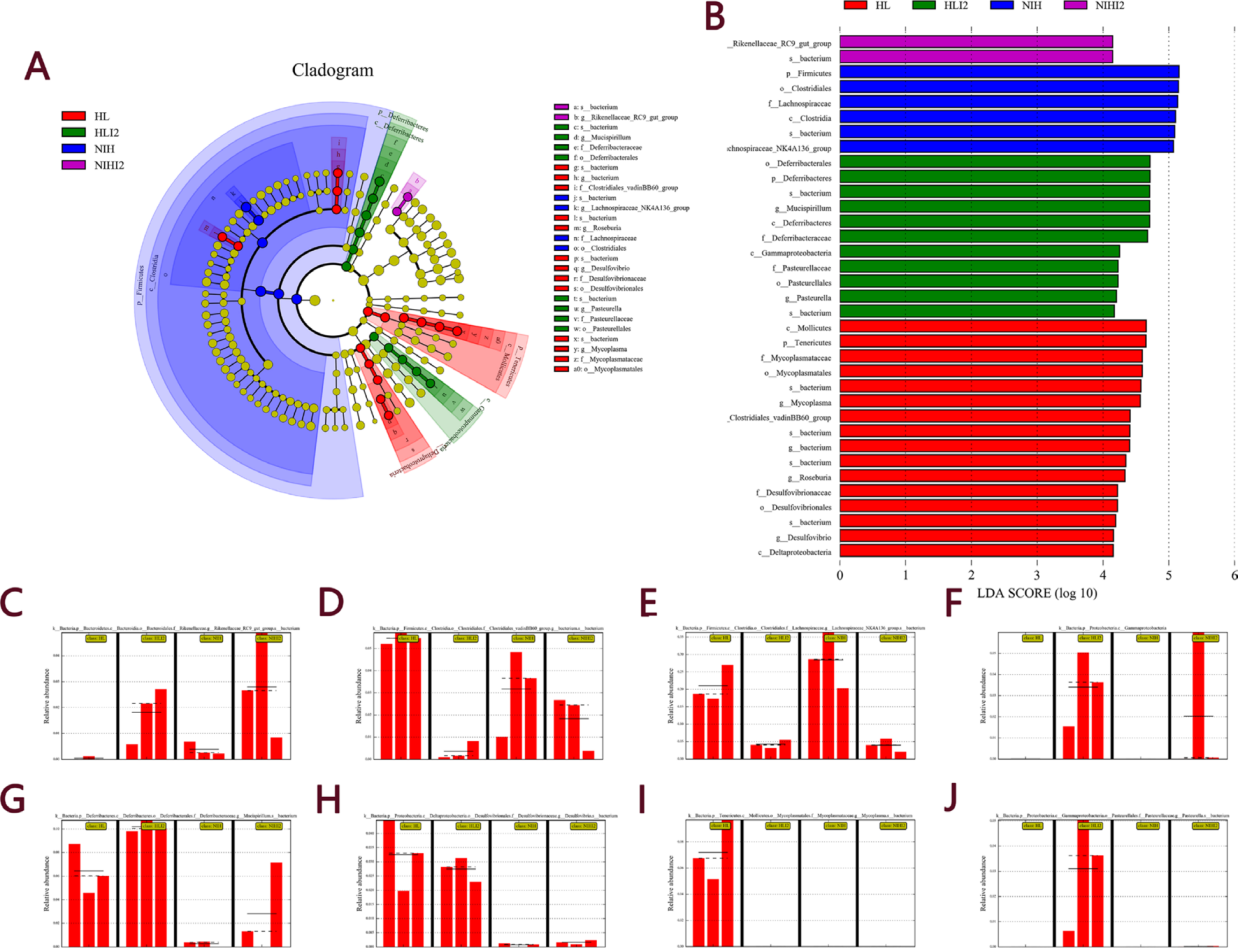
LEfSe analysis of the four groups (**A**) Cladogram of the four groups. (**B**) LDA score histogram. The relative abundance of (**C**) *Rikenellaceae*, (**D**) *Clostridiales*, (**E**) *Lachnospiraceae*, (**F**) *Gammaproteobacteria*, (**G**) *Mucispirillum*, (**H**) *Desulfovibrio*, (**I**) *Mycoplasma*, and (**J**) *Pasteurella*. Solid and dashed lines indicate mean and median, respectively.

The line discriminant analysis (LDA) effect size method was used to identify high-dimensional biomarkers in the intestinal microbiota among each group. The LDA score was set at 4.0, and different species for which the LDA score was >4 were considered significant biomarkers. The LDA value results were showed in Supplementary Table 2. The cladogram is shown in Figure 8A and LDA score distribution map is shown in Figure 8B. As shown in Figure 8C–8F, mice infected with *L. monocytogenes* showed an increase in the number of bacteria belonging to the *Rikenellaceae* family and *Gammaproteobacteria* class, and a decrease in bacteria belonging to the *Clostridiales* class and *Lachnospiraceae* family. Bacteria belonging to the *Mucispirillum* and *Desulfovibrio* genera were more highly abundant in HL mice (including HL normal and infected mice) than in NIH mice (Figure 8G, 8H). The *Mycoplasma* genus was present only in HL mice (Figure 8I), while the *Pasteurella* genus was present only in HL-infected mice (Figure 8J). Collectively, these results indicated that mice infected with *L. monocytogenes* can undergo changes in the intestinal microbiota composition. Changes in the abundance of these bacteria may alter lesion damages in the viscera. Raw data of our research was showed in Supplementary Table 3.

## DISCUSSION

Abnormalities in hair growth and hair follicle development in mutant mice are often accompanied by modulations in immunity [16, 17]. In this study, we used an *L. monocytogenes* infection model to evaluate the innate immunity of mutant mice. Previous data have shown that, compared to oral administration, the i.v. or i.p. administration of mice with a lethal dose of *L. monocytogenes* is 10–100 times more effective in causing infection [18]. Showing a marked tolerance, the situation with *L. monocytogenes* infection in humans is significantly different [5]. Studies have shown that fecal transplantation does not transfer either susceptibility or resistance to food-borne listeriosis in C57BL/6 and BALB/c/By mice [19]. Thus, depending on the habit of mice of eating excrement, the gut microbiota of two types of mice should converge after feeding in the same cage for 3–4 weeks [10, 11]. The four groups of mice were infected with *L. monocytogenes* at a density of 2 × 106 cfu. The innate immunity of NIH HL mice and the impact of gut microbial polymorphisms on *L. monocytogenes* infection were analyzed based on bacterial loads, histopathological lesions, and cytokine-expression levels.

The results showed that mutant HL mice are more susceptible to *L. monocytogenes* infection than NIH mice. Co-housing did not have a major impact on mice in terms of weight changes and survival. The liver and spleen are susceptible to *L. monocytogenes* infection. To investigate the infiltration of inflammatory cells, H&E- and Masson-stained liver and spleen sections of mice from all four groups were assessed. Consistent with previous results, inflammatory infiltration was obvious in the livers of HL mice compared to that in the livers of NIH mice, and the spleen tissues were severely damaged. Because the severity of infection in HL mice at the 2 × 106 cfu challenge dose confounded our ability to quantify the bacterial burden, and previous studies have shown that a peak bacillary burden is noted in the spleen and liver 3 days after infection [20], the density of microbial inoculum was decreased to 1 × 106 cfu to study the bacterial load. Our results showed that the bacterial burden in the spleen of HL and NIH mice was not significantly different after co-housing. Through analyses of bacterial loads and histopathological lesions, we found that changes in the gut microbiota after co-housing can partially relieve the spleen injury.

Monocytes and neutrophils play important roles in early host defense responses against microbial pathogens [11, 21–23]. We used an automated hematology analyzer to analyze the quantity and proportion of monocytes and neutrophils. In normal mice, the levels of both cell types were similar. However, after two days of infection, the number of monocytes and their proportion in white blood cells were significantly lower in HL mice than in NIH mice. This finding suggests that the decrease in monocytes may lead to increased susceptibility of HL mice to *L. monocytogenes* infection, compared to NIH mice. That the proportion of neutrophils in white blood cells increased may be due to the decrease in white blood cells. Gene mutations in HL mice (for example, mutations in the Hc gene) may block mRNA translation, resulting in inconsistent expression of inflammatory cytokines and proteins [5]. Further studies are required to confirm this possibility.

Mice infected with *L. monocytogenes* showed induction in bacteria belonging to the *Rikenellaceae* family and *Gammaproteobacteria* class, and decreased bacteria belonging to the *Clostridiales* class and *Lachnospiraceae* family. Studies have shown that bacteria belonging to the *Rikenellaceae* family increase in mice with acute liver failure [24]. Bacteria belonging to the *Gammaproteobacteria* class are dominant in aquatic environments, closely related to glucose metabolism and body health, but are not present in HL and NIH normal mice [25, 26]. The *Lachnospiraceae* family is related to glucose metabolism and its occurrence is decreased in obese mice [27]. The *Clostridiales* class can inhibit *L. monocytogenes* infection [28]. Reduced levels of uncultured *Clostridiales* are considered a signature of a compromised gut [29, 30]. Substantial reduction of *Clostridiales* in HL-infected mice may cause a serious infection. These findings indicate that changes in relative abundances of these bacteria not only result from *L. monocytogenes* infection, but were also the major cause of severe infection and death in infected mice.

In conclusion, our results showed that HL mice are more susceptible to *L. monocytogenes* than NIH mice. Mice infected with *L. monocytogenes* showed changes in the intestinal microbiota composition. The change in abundance of these bacteria may alter lesion damage in the viscera. In addition, we found that the *Mycoplasma* genus is a biomarker of HL mice, and the *Mucispirillum* and *Desulfovibrio* genera were more abundant in HL mice than in NIH mice.

## MATERIALS AND METHODS

### Ethics statement

Investigation has been conducted in accordance with the ethical standards according to national and international guidelines and has been approved by the authors’ institutional review board. Specifically, the animal experiments were performed in accordance with the requirements of the Experimental Animal Ethics and Welfare guidelines (Permit Number: 20160205).

### Animals and bacterial strains

Specific pathogen free-grade female NIH HL mice and normal NIH mice (2 or 6 weeks old) were provided by the Changchun Institute of Biological Products Co. Ltd. (SCXK-2016-0008). Two-week-old female NIH HL mice were co-housed with 2-week-old female NIH normal mice for 4 weeks. The bacterial strain used in this study was *L. monocytogenes* 10403S (serotype 1/2a). *L. monocytogenes* was cultured in brain heart infusion medium (Oxoid, Hampshire, England) and incubated in a shaker incubator at 37°C and 200 rpm.

### *In vivo* infection experiments

Animals were divided into four groups (HL, NIH, HL-co-housed with NIH, and NIH-co-housed with HL). Mice were infected (i.p.) with *L. monocytogenes* at a dose of 2 × 106 cfu, and the animals were weighed and monitored daily for mortality for up to 10 days. Survival was monitored daily for 10 days [11]. Mice were infected with *L. monocytogenes* at a dose of 1 × 106 cfu (i.p.). Bacterial loads in the liver and spleen were determined on day 3 after infection [31]. The density of bacteria used in subsequent infection experiments was 2 × 106 cfu.

### Histopathology

At 72 h post-infection, the liver, spleen, cecum, and colon were collected (*n* = 3), and paraffin sections were prepared and subjected to H&E and Masson staining. Images were acquired using an optical microscope (Olympus, Tokyo, Japan).

### Routine blood examination

At 0, 24, and 48 h post-infection, blood was collected from the orbital venous plexus using a capillary. Peripheral blood (20 μL) was diluted into 1 mL aliquots, which were analyzed with an automated hematology analyzer.

### Real-time PCR and ELISA

At 48 h post-infection, the liver and spleen were collected (*n* = 5). Detailed methods for RNA extraction and gene quantification are available in previous reports [4]. The primer pairs used for q-PCR amplification were listed in Table 1.

**Table 1:** Sequences of primers used in qPCR

Primer	Sequence (5′ to 3′)	Size (bp)	NCBI Entrez accession number
*TNF-α*F	CTTCTGTCTACTGAACTTCGGG	106 bp	NM_013693
*TNF-α*R	TGATCTGAGTGTGAGGGTCTG		
*IL-1β*F	CGGACCCATATGAGCTGAAAG	135 bp	NM_008361
*IL-1β*R	TCTTTCCTTTGAGGCCCAAG		
*IL-6*F	CAAAGCCAGAGTCCTTCAGAG	150 bp	NM_031168
*IL-6*R	GTCCTTAGCCACTCCTTCTG		
*Actb* (beta-actin)F	GTGGGAATGGGTCAGAAGG	149 bp	NM_007393.4
*Actb* (beta-actin)R	AGCTCATTGTAGAAGGTGTGG		

The liver and spleen were homogenized with PBS (4 mL/g), and lysates were centrifuged at 4500 × *g* for 30 min to collect the supernatant. Serum was also collected. ELISAs (Haling Biological, Shanghai, China) were performed to measure the protein-expression levels of inflammatory cytokines.

### Gut microbiota analysis

At 48 h post-infection, the cecum content was collected for the gut microbial analysis, and the samples were divided into four groups, namely, NIH normal mice (NIH), HL normal mice (HL), NIH-infected mice (NIHI2), and HL-infected mice (HLI2). Bacterial genomic DNA was extracted from frozen samples stored at −80°C. The V3 and V4 regions of the 16S rRNA gene comprising were amplified by PCR using specific bacterial primers (Table 2). High-throughput pyrosequencing of the PCR products was performed on an Illumina MiSeq platform at Biomarker Technologies Co. Ltd. (China). The raw paired-end reads from the original DNA fragments were merged using FLASH32 and assigned to each sample, according to the unique barcodes. QIIME [32] (version 1.8.0) UCLUST [33] software was used based on 97% sequence similarity. The tags were clustered into OTUs. The alpha diversity index was evaluated using Mothur software (version, v.1.30). To compare the diversity index among samples, the number of sequences contained in each sample was standardized. Analysis treasure included OTU rank, rarefaction, and Shannon curves, and the Shannon, Chao1, Simpson, and ACE indexes were calculated. For beta diversity analysis, heatmaps of RDA-identified key OTUs, PCoA [34], NMDS [35], and UPGMA were obtained using QIIME. The LDA-effect size (LEfSe) method was used for the quantitative analysis of biomarkers in each group. Briefly, LEfSe analysis, an LDA threshold >4, the non-parametric factorial Kruskal–Wallis sum-rank test, and the unpaired Wilcoxon rank-sum test were performed to identify the most differently abundant taxa [36, 37].

**Table 2:** Sequences of 16S rRNA (V3 + V4) primers

16S rRNA (V3 + V4) primers	Sequence (5′ to 3′)
F	ACTCCTACGGGAGGCAGCA
R	GGACTACHVGGGTWTCTAAT

### Statistical analysis

All data are shown as the mean ± SEM. Differences in the survival rates of animals were calculated by the log-rank Mantel–Cox test using GraphPad Prism software. In all other experiments, *p* values were calculated by the two-tailed Student’s *t*-test using GraphPad Prism software [11]. When F > 0.05, *p* values were calculated using the unpaired *t*-test with Welch’s correction.

## SUPPLEMENTARY MATERIALS FIGURE AND TABLES








