# Supplementary material for: Analyzing the innate immunity of NIH hairless mice and the impact of gut microbial polymorphisms on Listeria monocytogenes infection
Source: Oncotarget. 2017 Oct 25;8(63):106222–32. doi: 10.18632/oncotarget.22051 (PMC5739728; doi:10.18632/oncotarget.22051)
Supplement: Supplementary file 1 [file oncotarget-08-106222-s001.pdf]

Analyzing the innate immunity of NIH hairless mice and the impact of gut microbial polymorphisms on *Listeria monocytogenes* infection

SUPPLEMENTARY MATERIALS

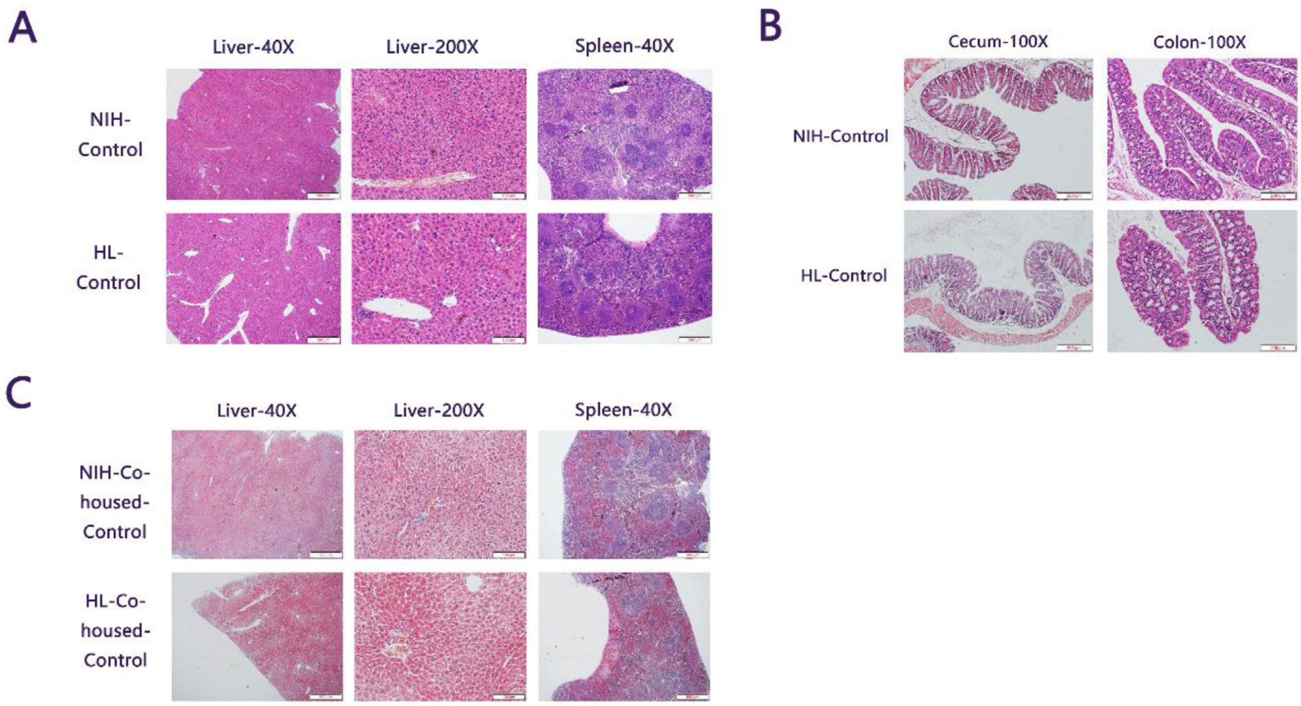

Supplementary Figure 1: (**A**) In NIH and HL mice, the liver and spleen were normal in morphology and structure, no inflammatory cell infiltration was found in the liver and the boundaries between red pulp and white pulp were obvious. (**B**) In HL-Co-housed and NIH-Co-housed mice, the liver and spleen were normal in morphology and structure, no inflammatory cell infiltration was found in the liver and the boundaries between red pulp and white pulp were obvious. (**C**) The morphology of cecum and colon in NIH and HL mice.

Supplementary Table 1: Sample sequence data. See Supplementary_Table_1

Supplementary Table 2: LDA value list. See Supplementary_Table_2

Supplementary Table 3: Raw data. See Supplementary_Table_3
